# Expanding the Role of the Histone Lysine-Specific Demethylase LSD1 in Cancer

**DOI:** 10.3390/cancers11030324

**Published:** 2019-03-07

**Authors:** Barbara Majello, Francesca Gorini, Carmen Daniela Saccà, Stefano Amente

**Affiliations:** 1 Department of Biology, University of Naples ‘Federico II’, 80126 Naples, Italy; carmendaniela.sacca@unina.it; 2 Department of Molecular Medicine and Medical Biotechnologies, University of Naples, ‘Federico II’, 80131 Naples, Italy; francesca.gorini@unina.it (F.G.); stefano.amente@unina.it or stamente@unina.it (S.A.)

**Keywords:** LSD1, epigenetics, histone demethylase, cancer, lncRNAs

## Abstract

Studies of alterations in histone methylation in cancer have led to the identification of histone methyltransferases and demethylases as novel targets for therapy. Lysine-specific demethylase 1 (LSD1, also known as KDM1A), demethylates H3K4me1/2, or H3K9me1/2 in a context-dependent manner. In addition to the well-studied role of LSD1 in the epigenetic regulation of histone methylation changes, LSD1 regulates the methylation dynamic of several non-histone proteins and participates in the assembly of different long noncoding RNA (lncRNA_ complexes. LSD1 is highly expressed in various cancers, playing a pivotal role in different cancer-related processes. Here, we summarized recent findings on the role of LSD1 in the regulation of different biological processes in cancer cells through dynamic methylation of non-histone proteins and physical association with dedicated lncRNA.

## 1. Introduction

Considerable efforts have been placed to understand how changes in the epigenome contribute to cancer progression [1,2]. Accordingly, enzymes that control epigenetic marks have been investigated as potential targets for cancer therapy [3,4]. It is widely accepted that chromatin modifiers play an important role in tumorigenesis [1,2,3,4]. Histone acetylation generally correlates with transcriptional activation [5], while histone methylation serves as activating or repressive transcriptional marker, depending on the location and degree of residue methylation [6]. Methylation may occur on both arginine and lysine residues and creates docking sites for recognition and binding of so-called reader proteins [7].

The demethylase 1 (LSD1/KDM1A) lysine-specific demethylase 1 is an epigenetic enzyme whose overexpression is correlated with poor prognosis in a variety of cancers [8,9,10,11,12] and has been proposed as therapeutic target [13]. LSD1 demethylates mono- or di-methyl-lysine 4 or 9 of histone H3 (H3K4me1/me2 and H3K9me1/me2, respectively) depending on its interacting partners [14,15,16,17,18,19,20].

LSD1 (KDM1A/AOF2), with LSD2 (KDM1B/AOF1), is a member of the flavin-dependent LSD/KDM1 demethylase protein family. Both enzymes contain the Swi3p, Rsc8p, and Moira (SWIRM) and amine oxidase (AO) domains. Structurally, LSD1 contains a coiled-coil Tower domain protruding from the AO domain responsible for interaction with its co-factors, while LSD2 possesses the amino-terminal zinc finger element that is necessary for LSD2 binding to its methylated substrate [21]. Although LSD1 and LSD2 share significant homology and both enzymes demethylate lysine 4 on histone 3 in a flavin adenosine dinucleotide (**FAD**)-dependent manner, the two enzymes have distinct functions, affecting different steps of the gene transcription. Genome-wide chromatin-immunoprecipitation studies have shown that LSD1 binds to the enhancer and promoter regions of genes [22,23]; in contrast, LSD2 associates primarily with the body regions of actively transcribed genes [21,24].

A large number of studies have highlighted the pivotal role of LSD1 in several cellular processes in normal and cancer cells such as control of stemness, differentiation [12,22,23], cell motility, epithelial-to-mesenchymal transition [10,25,26], autophagy [9,27], senescence [28], neurodegenerative diseases [29,30], and metabolism [31]. To achieve these widespread biological functions, LSD1 is a component of different multi-protein complexes and its association with over 60 gene regulatory proteins has been demonstrated [14,15,32,33,34].

While the role of LSD1 as an epigenetic master regulator of the transcription (co-activator or co-repressor) has been well explored, its function in the methylation dynamics of several non-histone proteins and in the assembly of different long noncoding RNA (lncRNA) complexes is an emerging field.

This review will be focused on the increasing recent evidences that characterize LSD1 as a functional regulator of non-histone proteins and lncRNAs in the biology of cancer cells.

## 2. LSD1 as Transcriptional Coregulator

**Figure 1:**
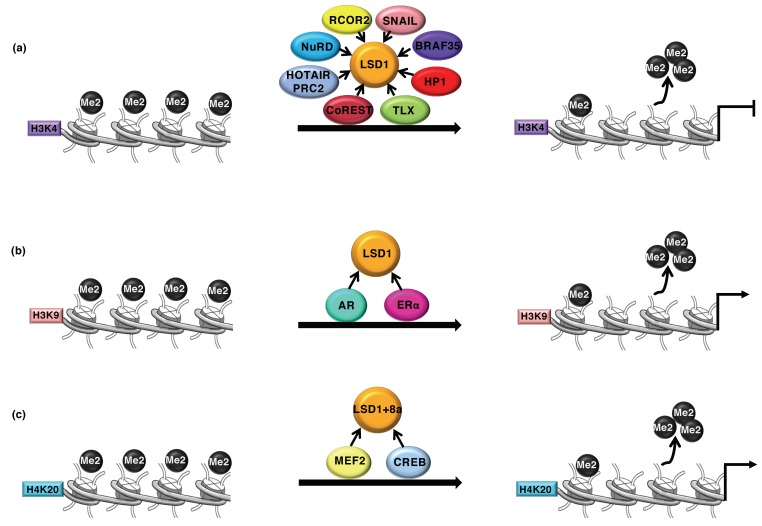
(**a**) Demethylase 1 (LSD1) is recruited at the target gene by a wide number of transcription factors (indicated in figure) where it promotes the transcriptional repression through demethylation of the H3K4me2 activation mark. (**b**) LSD1 demethylates the repressive mark H3K9me2 as coactivator with the androgen or estrogen receptor. (**c**) LSD1+8a, a neuronal-specific isoform, catalyzes the demethylation of the repressive mark H4K20me2, by interacting with CREB and MEF2.

LSD1 was first isolated as the interacting partner of the CoREST (RCOR1) transcription repressor complex [35] and the histone deacetylase HDAC1/2 [36]. In complexes with CoREST and NuRD (Nucleosome Remodeling and Deacetylase), LSD1 demethylates monomethyl and dimethyl histone H3 lysine 4 (H3K4me1/me2), which mark an active chromatin transcription state [37,38,39,40,41,42] (Figure 1a).

LSD1 represses the transcription process and interacts with members of the SNAG domain of transcription factors such as SNAIL1/2 and GFI1/B. The LSD1-CoREST complex, through interaction with the SNAG domain of SNAIL, is recruited to the E-cadherin (CDH1) promoter to remove methyl groups on lysine 4 of histone H3 resulting in repression of its expression [43].

Despite its well-reported activity as transcription repressor, LSD1 is also a co-activator of the androgen (AR) and estrogen (ER) receptor-dependent transcription. The interaction between LSD1 and AR or ER nuclear receptors directly or indirectly modify its substrate specificity through demethylation of the repression-associated H3K9me1/2 mark [15,16] (Figure 1b). Moreover, it has been proposed that LSD1 recruitment by ER or c-Myc on target genes triggers DNA oxidation and recruitment of base excision repair enzymes that favor chromatin looping of transcriptional activation [14,15].

Different behavior and tissue specificity have been addressed to the splicing variant of LSD1 (LSD1+8a) containing four additional amino acids (exon 8a) within the AO domain [44]. The presence of exon 8a generates a docking site for supervillain (SVIL) converting LSD1 into an H3K9 demethylase in neuronal differentiation [45]. Moreover, LSD1+8a has also been described to promote the transcription of neuronal-regulated genes by removing H4K20me2 (Figure 1c) [46]. Intriguingly, LSD1+8a activity is required for memory and spatial learning [46].

Finally, the LSD1 protein is target of different post-translational modifications that alter its activity or stability expanding its possibility to exert different roles in transcription regulation [8,47]. LSD1 phosphorylation at serine 112 by the PKCα (Protein kinase C alpha) is crucial for its recruitment on E-cadherin promoter [48]. Moreover, LSD1 acetylation by the acetyltransferase MOF is specific of epithelial cells and seems to be critically involved in LSD1-induced epithelial to mesenchymal transition (EMT) [49].

## 3. LSD1 and Cancer

LSD1 has been proposed as a druggable target in cancer for a large number of observations reporting its high expression levels in poor prognosis of prostate, lung, brain, and breast cancers, as well as in certain hematologic malignancies [13]. Accordingly, different LSD1 inhibitors are currently in clinical trial studies [13,50]. In addition, it has been recently reported that high rates of germline truncating and missense LSD1 mutations occur in multiple myeloma (MM), suggesting that LSD1 is an autosomal dominant MM predisposition gene [51]. It is plausible that LSD1, through interaction with different factors, might exert distinct molecular mechanisms in various tumor types. Nevertheless, LSD1 plays important role in cellular processes shared by different cancers such as EMT, as comprehensively described in recent reviews [26,27].

Notably, the paradigmatic role of LSD1 in cancer, based on its histone demethylation capacity, has been questioned in two recent reports [22,52]. LSD1 is significantly expressed in less differentiated subtypes of acute myeloid leukemia (AML), and several data support the oncogenic potential of LSD1 in AMLs, and in particular, its capacity to sustain leukemia stem cells [22]. Intriguingly, in a recent work, Maiques-Diaz et al. [22] remarkable discovered that LSD1 promote the differentiation in AML through mechanisms that are histone demethylases activity independent. Specifically, inhibition of LSD1 results in the functional inactivation of transcription repressor GFI1 with disruption of GFI1-LSD1-RCOR1 interaction [22,53].

In prostate cells, Sehrawat et al. [52] showed that LSD1 promotes survival of castration-resistant prostate cancer cells, independently of its demethylase function. Inactivation of LSD1 increased the expression of a subset of androgen receptor (AR) target genes. Furthermore, the authors suggest that the effects of LSD1 on cell survival are explained in part by the activation of a lethal prostate cancer gene network in collaboration with the binding protein ZNF217 [52].

Whether such demethylase-independent mechanisms are conserved in other malignancies needs further investigations.

## 4. Non-Histone Substrates of LSD1

**Figure 2:**
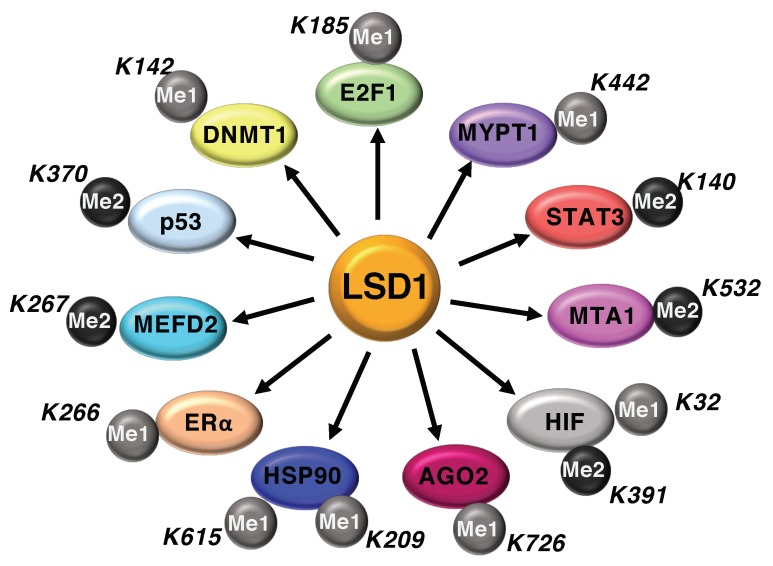
LSD1 regulates methylation dynamics of non-histone proteins.

Although LSD1 was originally identified as histone lysine demethylase, several reports highlight the role of LSD1 to regulate the activity of non-histone proteins through LSD1-dependent demethylation mechanisms. Indeed, LSD1 participates to the methylation/demethylation dynamics on specific lysine residues of several non-histone proteins, including, p53, DNMT1, STAT3, E2F1, RB1, MEFD2, MTA1, ERα, HSP90, HIF-1α, and more recently AGO2 (Figure 2) [12]. The demethylase activity of LSD1 alters both the function and stability of the aforementioned proteins. Specifically, LSD1 enhances STAT3 and p53 functional activity without changes in their expression levels, whereas it destabilizes the protein MYPT1 and stabilizes E2F1, MEFD2 and HIF-1α proteins.

Understanding how the demethylation of lysine residues of non-histone proteins by LSD1 influences different cellular processes is an emerging field in the biology of cancer cells.

### 4.1. LSD1 Modulates the Activity of Cell-Cycle Regulators

**Figure 3:**
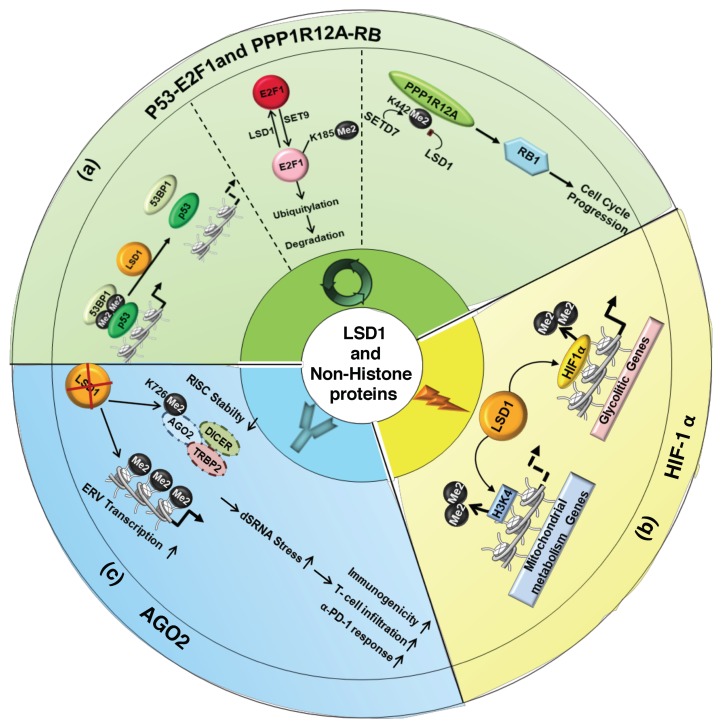
LSD1-dependent demethylation regulates activity of non-histone proteins. Effects of LSD1-mediated demethylation of non-histone proteins p53-E2F1 and PPP1R12A (**a**), HIF-1α (**b**), and AGO2 (**c**).

The first discovered target of LSD1 demethylation was p53 [54], one of the most important tumor suppressor proteins. p53 controls different cellular stress signals, in particular, it activates transcription targets involved in the control of cell cycle and apoptosis. After DNA Damage, p53 is phosphorylated by ATM (Ataxia Telangiectasia Mutated) and induces p21 transcription with consequent cell cycle arrest. p53 is regulated by MDM2 (Murine Double Minute 2), which binds p53 and determines its proteasome dependent degradation. Moreover, there is a dual relationship between MDM2 and p53. Indeed, MDM2 itself is positively regulated by p53, which functions as a transcriptional factor of the mdm2 gene. p53 is methylated by SMYD2 on lysine 370 (K370me1/me2); this modification is required for its association with the co-activator p53 Binding Protein 1 (53BP1) [54,55]. LSD1 demethylates p53 K370me1/me2, thus inhibiting its binding to DNA and its pro-apoptotic activity (Figure 3a) [54].

LSD1 also regulates DNA damage-induced cell death demethylating E2F1 lysine 185 (K185) in p53-deficient cancer cells [56]. LSD1 maintains unmethylated E2F1 in the cells, which can be upon DNA damage hyperacetylated and phosphorylated by PCAF and CHK2, respectively. E2F1 hyperacetylated and phosphorylated cannot be methylated on K185 by Set9 and, escaping degradation, accumulates in the cells to promote apoptosis (Figure 3a). Finally, the demethylation activity of LSD1 promotes DNA damage-induced cell death by stabilizing E2F1 in p53-deficient tumor cells, whereas it inhibits apoptosis repressing p53 transcriptional activity [56,57].

The role of LSD1 in the cell cycle is also supported by its activity toward the retinoblastoma protein (RB1) [58]. Cell-cycle phosphorylation dynamics of RB1 is required for proper control of cell cycle progression and low levels of RB1 restrict cells ability to replicate DNA with cell division arrest in the G1 phase. RB1 is dephosphorylated by the phosphatase PPP1R12A (also known as MYPT1). However, PPP1R12A lysine 442 is methylated by the histone lysine methyltransferase SETD7 and demethylated by LSD1. LSD1-mediated demethylation of PPP1R12A enhances its polyubiquitylation and proteasome degradation and therefore increases the levels of phosphorylated RB1 (Ser 807/811) promoting cell cycle progression (Figure 3b) [58].

Taken together, these findings support the role of LSD1, as a modulator of the methylation/demethylation dynamic of RB1, into the cell cycle regulatory mechanism and human carcinogenesis.

### 4.2. LSD1-Mediated Demethylation Regulates HIF-1α Function

Relevant in cancer progression is the functional interaction between LSD1 and HIF-1α (Figure 3c) [59,60,61,62]. HIF-1α plays a crucial role in the development of aggressive cell phenotype (through stimulation of angiogenesis, epithelial-mesenchymal transition, and invasion) and in glycolytic and mitochondrial metabolism interplay. Among the various post-translation modifications, HIF-1α is methylated by the SET7/9 histone methyltransferase at lysine 32 residue (K32) [60]. Moreover, SET9 methylases the HIF-1α lysine 391 (K391) [62], and that determines the HIF-1α ubiquitin-mediated protein degradation. HIF-1α K32 and K391 methylated residues are demethylated by LSD1 [60,62]. In addition, HIF-1α is hydroxylated at proline 402 and 564 residues (P402/564) and such modifications are required for methylation of K391. HIF-1α hydroxylation, as well as its methylation, is directly suppressed by LSD1. Thus, LSD1 prevents HIF-1α ubiquitination and degradation by inhibiting both hydroxylation and methylation [62]. Moreover, LSD1 further controls HIF-1α protein stability, in an FAD-dependent manner, demethylating the lysine 271 residue (K271) of RACK1 protein, a component of the HIF-ubiquitination complex. The demethylation of RACK1 impairs its binding to HIF-1α and suppresses RACK1-mediated HIF-1α degradation [61].

LSD1, through HIF-1α stabilization, induces genes involved in glycolysis in cancer cells [31]. LSD1 directly represses genes involved in mitochondrial metabolism demethylating histones H3K4 (Figure 3c).

LSD1 plays a key role in the reprogramming of cancer metabolism inducing the shift from oxidative to glycolytic metabolism, maintenance of redox homeostasis, and cell survival.

### 4.3. LSD1 and Immunogenicity

Recent studies discovered a novel function of LSD1 in modulating the tumor immunogenicity. Work from Sheng’s [63] laboratory has shown that LSD1, through demethylation of AGO2 (Protein Argonaute 2) has a crucial role in the suppression of anti-tumor immunity and tumor immunogenicity [63].

AGO2 is a core element of the RNA-inducing silencing complex (RISC) and directly initiates degradation of target RNAs through its catalytic activity. AGO2 protein stability depends on LSD1 demethylation of its lysine 726 residue (K726). In breast and melanoma cells, it has been demonstrated that LSD1 inhibition reduces RISC activity, via methylation-dependent de-stabilization of AGO2, and concomitantly increases endogenous retroviral (ERV) expression, leading to dsRNA stress (Figure 3c) [63]. RNA stress determines activation of type 1 interferon (IFN) response that consequently sensitizes tumors to T cell immunity and T cell infiltration into melanoma tumors [63]. Moreover, LSD1 ablation in these tumor cells results in the upregulation of PD-(L)1, the programmed death-ligand 1, which might compromise the anti-tumor effect of T cells infiltration. Indeed, PD-(L)1, expressed on cancer cells, binds PD-1 (programmed death-1), expressed on the surface of immune cells, and forms a biochemical “shield” protecting tumor cells from being destroyed by the immune system. Conversely, in LSD1-KO tumors, upregulation of PD-1 renders refractory tumor responsive to anti-PD-1 therapy [63]. Thus, LSD1 inhibition and concomitant PD-1 blockade have shown a synergistic effect in the reduction of tumor growth [63].

Very recently, Qin et al. [64] reported the role of LSD1 in regulation of breast tumor immunogenicity. LSD1 inhibition increases the expression of key immune checkpoint regulators such as CCL5, CXCL9, and CXCL10 and effector T cell chemokines, which in turn increases CD8+ T cell tumor infiltration and improves the efficacy of immunotherapy [64]. This study further supports the potential role of LSD1 inhibition in cotreatment with immunotherapy as a novel management strategy for poorly immunogenic breast tumors.

In conclusion, LSD1 inhibition could be instrumental to convert “cold” tumors (resistant to PD-1 blockade) in “hot” tumors (responsive to PD-1 therapy) and provides a means to target LSD1 to increase the efficacy of immunotherapy of poor immunogenic cancers.

## 5. LSD1 and lncRNA

Long noncoding RNAs (lncRNAs) are emerging classes of regulatory RNAs that operate as pivotal modulators in different molecular processes such as gene regulation, maintenance of genome packaging, chromatin dynamics, and cell differentiation. Recent studies propose lncRNAs as players in regulation of gene expression in immune cells and in a variety of human diseases, including cancers. Indeed, lncRNAs are involved in cancer development through different pathways; specifically, their deregulation induces cancer cells to initiate tumor growth and metastatic processes [65]. Various evidences have indicated that several lncRNA contribute to cancer cell phenotypes through the interaction with chromatin-modifying agents and proteins, such as the polycomb repressive complex 2, LSD1, CoREST, and SMCX.

**Figure 4:**
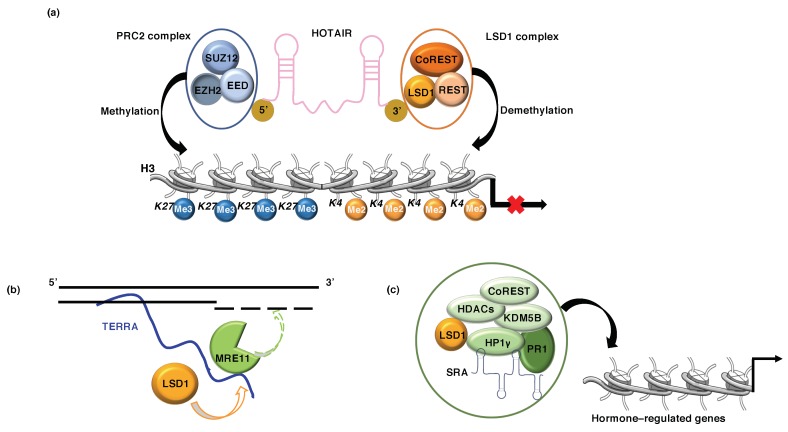
(**a**) HOTAIR mediates target gene silencing; it binds to PRC2 complex (EZH2, SUZ12, EEDs) and LSD1 complex (CoREST/REST) through binding at 5′ and 3′ ends, respectively, regulating trimethylation of H3K27me3 and demethylation of H3K4me2. (**b**) TERRA RNA modulates telomeric gene silencing through recruitment of LSD1 on uncapped telomeres, reinforcing the interaction between LSD1 and MRE11. (**c**) SRA interacts with the repressive complex LSD1-HP1γ-HDAC1/2-CoREST (LSD1.com) and brings this complex on target chromatin, promoting aberrant hormone-regulated gene expression.

Here, we highlight the role of LSD1 through functional and physical interaction with a plethora of lncRNAs (Table 1 and Figure 4).

### 5.1. LSD1 and lncRNA HOTAIR

HOTAIR (HOX antisense intergenic RNA) is an lncRNA originally discovered by Rinn et al. [66] that acts as a modular scaffold to induce gene silencing by regulating chromatin dynamics [67]. HOTAIR regulates cell cycle progression and its expression is correlated with poor prognosis in breast, pancreatic, and colon cancer patients [68]. The mechanism by which HOTAIR regulates gene expression has been elucidated; it has been found that HOTAIR acts as scaffold of various different complexes involved in different types of human cancers. Its epigenetic role is exerted through its association with two histone modifiers: while the 5′ domain of HOTAIR binds the histone methylase PRC2, the 3′ domain scaffolds the histone demethylase LSD1. Such complexes are recruited to target gene loci resulting in transcription repression via H3K27-trimethylation (PRC2 activity) and H3K4-demethylation (LSD1 activity) (Figure 4a) [68,69].

It has been found that in pancreatic cancers, HOTAIR-mediated gene repression is both PRC2-dependent [70] and independent [71]; however, in glioma cells, HOTAIR regulates cell cycle progression predominantly via the PRC2 axis (EZH2-dependent) [70]. Moreover, in breast cancers, HOTAIR functions as scaffold for LSD1 and HBXIP (Mammalian hepatitis B X-interacting protein) on c-Myc target genes [72]. HBXIP is found highly expressed in breast cancer and it generally regulates the expression of genes involved in cancer growth and metastasis interacting with different transcription factors (TF-II, STAT3, E2F1, SP1, and c-Myc). In particular, the oncoprotein HBXIP interacts directly with c-Myc leading to the recruitment of HBXIP-HOTAIR-LSD1 complex on the E-box of c-Myc target genes and determines their activation [72].

These findings reveal that targeting HOTAIR might be an innovative therapeutic strategy aimed to disrupt HBXIP-HOTAIR-LSD1 function.

### 5.2. LSD1 and lncRNA TERRA

TERRA (Telomeric Repeat containing RNA) is an lncRNA which is transcribed from subtelomeres toward chromosome ends by RNA Polymerase II, where it remains partly associated with telomeric chromatin and plays a key role in telomere length control. Furthermore, TERRA modulates telomeric gene silencing through recruitment of the demethylase (LSD1) and the methyltransferase (SUV39H1) that regulate H3K4/K9 methylation patterns [73,74,75,76]. TERRA binds the SWIRM/AOL-N-terminal domain of LSD1, this interaction stabilizes LSD1 binding at dysfunctional chromosome; subsequently, RNA-LSD1 interaction activates MRE11, which through its nuclease activity trims the 3′G overhangs at uncapped telomeres. MRE11 acts in sensing dysfunctional telomerase by promoting ATM-dependent DNA-damage signaling (Figure 4b) [77].

These findings highlight that the interaction between LSD1 and lncTERRA controls the telomere length with a strong impact in aging and cancer [77,78]. It is pertinent to note that TERRA is overexpressed in different types of cancer. TERRA accumulates and forms TERRA foci (TERFs) in highly proliferating progenitors and in human medulloblastoma tissue [79], suggesting that TERFs may provide a novel and sensitive biomarker for telomere dysfunction in cancer.

### 5.3. LSD1 and lncRNA SRA

In breast cancer cells the lncRNA SRA (Steroid Receptor RNA Activator), originally identified in human B-lymphocyte [80], plays a scaffolding role bringing together the repressive complex LSD1-HP1γ-HDAC1/2-CoREST-KDM5B, referred as HP1 γ-LSD1 complex [81,82]. In breast cancer cells, SRA can function as central platforms for the assembly of different molecular components. It has been demonstrated that SRA, interacting with HP1 γ-LSD1 complex and anchoring this repressive complex on target chromatin, promotes aberrant progesterone-regulated gene expression and affects hormone-dependent proliferation and apoptosis (Figure 4c) [81,82]. Recently, it has also been found that the HP1γ-LSD1 complex is recruited, through interaction with the ATP-dependent remodeling protein BRG1, by ligand-activated progesterone receptor (PR) to transcriptionally repressed genes involved in cell proliferation [83].

These findings suggest the targeting of the HP1γ-LSD1 complex for the management of hormone-dependent cancers.

### 5.4. LSD1 and the Oncogenic lncRNAs

A growing list of studies has underlined the scaffolding role of the lncRNAs for epigenetic enzymes as LSD1 and EZH2 to form ribonucleoprotein complexes capable to repress the transcription of tumor suppressor gene. Then, the lncRNA-LSD1/EZH2 complexes exert oncogenic function and promote tumor development and progression (Table 1).

In gastric cancer cells, three lncRNAs (LINC00673, FOXD2-AS1, HOXA11-AS) have been identified to interact with LSD1 and EZH2. In particular, the lncRNAs LINC00673 [84] and FOXD2-AS1 [85], in association with LSD1 and EZH2 repress, LAST2/KLF2 and EphB3 tumor suppressors, respectively. The HOXA11-AS (homeobox HOX A11 antisense mapping at the HOXA gene cluster) lncRNA scaffolds the chromatin modification factors PRC2, LSD1, and DNMT1 to regulate PRSS8 and KLF2 at transcriptional levels [86]. Another member of the HOXA gene cluster, HOTTIP, through binding to LSD1 and EZH2, represses LAST2 expression in renal cell carcinoma [87].

In non-small cell lung cancer (NSCLC) two lncRNAs have been found to exert oncogenic function interacting with EZH2 and LSD1: Linc01133, which regulates the transcription of KFL2, p21 and E-cadherin controlling cell proliferation, migration and invasion as well as apoptosis [88]; and FEZF1-AS that epigenetically represses the expression of E-cadherin enhancing EMT process [89].

In pancreatic cancer cells (PC) and colorectal cancer tissue (CRC), HOXA cluster antisense RNA2 lncRNA (HOXA-AS2) is involved in cell growth forming a complex with LSD1 and EZH2 [90,91]. The knockdown of HOXA-AS2 blocks the cell cycle transition and then provokes apoptosis, repressing p21 and KFL2 transcription. In PC cells, the lncRNA IRAIN forms a complex with LSD1 and EZH2, suppressing apoptosis and promoting proliferation [92].

Finally, in cholangiocarcinoma (CCA) and in osteosarcoma tumors (OS), the SPRY4-IT1 [93] and FOXP4-AS1 [94] lncRNAs, have been shown to play a role in tumor growth. Mechanistically, SPRY4-IT1 recruits EZH2 and LSD1 or DNMT1 to KLF2 and LATS2 promoter regions inducing epigenetic silencing [93]. Conversely, FOXP4-AS1 lncRNA, in complex with LSD1 and EZH2, sustains OS cell growth by repressing LATS1 transcription [94].

Collectively, these findings highlight the oncogenic function of specifics lncRNAs, suggesting that they may represent novel targets for therapy in the several human cancer diseases.

## 6. Conclusions

LSD1 constitutes a promising epigenetic target to treat different malignancies. Accordingly, many clinical trials using diverse LSD1 inhibitors are currently underway [13,50]. However, despite significant progress in understanding structural-functional aspects of LSD1 function, the molecular mechanisms underlying its involvement in cancer biology are not firmly established.

LSD1 was originally defined as a histone ‘eraser’ capable to demethylate H3K4m1/2 and H3K9me1/2 [14,15,16,17,18,19,20]. Chromatin immunoprecipitation studies established that LSD1 is present at enhancers and transcriptional start sites (TSS) of a large number of Polymerase-II-dependent genes [22,23]. The current paradigm suggests that LSD1 is recruited at enhancer chromatin regions thought interaction with multi-protein complexes containing a specific DNA-binding transcription factor. Accordingly, it is well established that LSD1 is present in a large number of transcription factors complexes affecting several biological functions depending upon the specific complex in which LSD1 is present [14,15,16,17,18,19,20,22,23,25,34,35,43,54,55,56,57,58,59,60,61]. The current assumption is that LSD1 is recruited at regulatory chromatin sites thought association with dedicated DNA-binding transcription factors. A number of evidences have clearly demonstrated that LSD1 can be recruited at specific targets via formation of riboprotein complexes containing lncRNAs, which act as modular scaffold of different histone modulators, including LSD1, in different type of human cancers.

An additional expanding role of LSD1 in cancer comes from the discovery of the capability of LSD1 to regulate activity of non-histone proteins as a result of LSD1-dependent demethylation mechanisms. Thus, LSD1 affects the methylation/demethylation dynamics on specific lysine residues of several non-histone proteins. It is conceivable that the multifaceted functions of LSD1 might account for its role in several different cellular processes in normal and cancer cells [66,67,68,69,70,71,72,73,74,75,76,77,78,79,80,81,82,83,84,85,86,87,88,89,90,91,92,93,94].

It is important for future studies to consider the methylation-demethylation dynamics of LSD1 histone substrates in order to establish the LSD1 function to epigenetic plasticity, and how LSD1 could contribute to cancer-associated epigenomic changes. In addition, the capability of LSD1 to modulate the methylation status of several important non-histone substrates, involved in cellular homeostasis, extends the role of LSD1 in several cellular processes which are deregulated in cancer.

We expect that a deeper characterization of the molecular functions of LSD1 in cancers will pave the way to the development of novel strategies for innovative treatment of cancer.

## Figures and Tables

**Table 1:** List of long noncoding RNAs (lncRNAs) that interact with LSD1 in several cancers.

lncRNA	Tumor	Reference
HOTAIR	Breast, Pancreatic and Glioblastoma	[68,69,70,71,72]
TERRA	Cervical	[73,74,75,76,77,78,79]
SRA	Breast	[80,81,82,83]
Linc00673	Gastric	[84]
FOXD2-AS1	Gastric	[85]
HOXA11-AS	Gastric	[86]
HOTTIP	Renal Cell Carcinoma	[87]
Linc01133	Non-small cell lung	[88]
FEZF1-AS	Non-small cell lung	[89]
HOXA-AS2	Pancreatic and Colorectal	[90,91]
IRAIN	Pancreatic	[92]
SPRY4-IT1	Cholangiocarcinoma	[93]
FOXP4-AS1	Osteosarcoma	[94]
